# Correction: Lesgards et al. Do Long COVID and COVID Vaccine Side Effects Share Pathophysiological Picture and Biochemical Pathways? *Int. J. Mol. Sci.* 2025, *26*, 7879

**DOI:** 10.3390/ijms26199513

**Published:** 2025-09-29

**Authors:** Jean-François Lesgards, Dominique Cerdan, Christian Perronne

**Affiliations:** 1 Independent Researcher, 13397 Marseille, France; 2 Independent Researcher, 33000 Bordeaux, France; 3 Infectious Diseases Department, University Hospital Raymond Poincaré, APHP, Université de Versailles Saint-Quentin-Paris Saclay, 92380 Garches, France

In the original publication [1], there were mistakes in the references [28,30,31,50]. The authors found a few mistakes in the table, this will apply only to the references in Table 1, which should be replaced with new references. In addition, reference [50] cited in the Section 5 should also be replaced with new reference [31]. The correct references that replaced references [28,30,31,50] are as follows:

28. Seighali, N.; Abdollahi, A.; Shafiee, A.; Amini, M.J.; Teymouri Athar, M.M.; Safari, O.; Faghfouri, P.; Eskandari, A.; Rostaii, O.; Salehi, A.H.; et al. The global prevalence of depression, anxiety, and sleep disorder among patients coping with Post COVID-19 syndrome (long COVID): A systematic review and meta-analysis. *BMC Psychiatry* **2024**, *24*, 105.
30. Balasubramanian, I.; Faheem, A.; Padhy, S.K.; Menon, V. Psychiatric adverse reactions to COVID-19 vaccines: A rapid review of published case reports. *Asian J. Psychiatr.* **2022**, *71*, 103129.
31. Ozturk, M.; Kumova Guler, D.; Oskan, E.E.; Onder, F. Long-Term Effects of COVID-19 on Optic Disc and Retinal Microvasculature Assessed by Optical Coherence Tomography Angiography. *Diagnostics* **2025**, *15*, 114. https://doi.org/10.3390/diagnostics15010114
50. Franzblau, L.E.; Mauskar, M.; Wysocki, C.A. Macrophage Activation Syndrome Complicated by Toxic Epidermal Necrolysis Following SARS-CoV-2 mRNA Vaccination. *J. Clin. Immunol.* **2023**, *43*, 521–524.

For references [30] and [31] in Section 5, number were change to [272] and [268], respectively. The correct reference that replaced references [30,31] are as follows:

268. Hou, Y.; Gu, T.; Ni, Z.; Shi, X.; Ranney, M.L.; Mukherjee, B. Global Prevalence of Long COVID, its Subtypes and Risk factors: An Updated Systematic Review and Meta-Analysis. *medRxiv* **2025**. https://doi.org/10.1101/2025.01.01.24319384.
272. Ogar, C.K.; Quick, J.; Gilbert, H.N.; Vreman, R.A.; Mantel-Teeuwisse, A.K.; Mugunga, J.C. Adverse Events to SARS-CoV-2 (COVID-19) Vaccines and Policy Considerations that Inform the Funding of Safety Surveillance in Low- and Middle-Income Countries: A Mixed Methods Study. *Drug Saf.* **2023**, *46*, 357–370. https://doi.org/10.1007/s40264-023-01279-3.

Therefore, the reference numbers in the Section 5 were re-numbered. With this correction, the order of some references has been adjusted accordingly. The authors state that the scientific conclusions are unaffected. This correction was approved by the Academic Editor. The original publication has also been updated.

## References

[1] Lesgards J.-F., Cerdan D., Perronne C. (2025). Do Long COVID and COVID Vaccine Side Effects Share Pathophysiological Picture and Biochemical Pathways?. Int. J. Mol. Sci..

